# Association of hemoglobin variability with the risk of cardiovascular disease: a nationally representative retrospective cohort study from South Korea

**DOI:** 10.1038/s41598-023-28029-w

**Published:** 2023-02-07

**Authors:** Won Jung Lee, Seulggie Choi, Sang Min Park, Gyeongsil Lee, Jooyoung Chang, Yun Hwan Oh, Joung Sik Son, Kyae Hyung Kim, Soo Jung Choi

**Affiliations:** 1 Department of Medicine, Seoul National University College of Medicine, Seoul, South Korea; 2 Department of Biomedical Sciences, Seoul National University Graduate School, 71 Daehak-Ro, Jongno-Gu, Seoul, South Korea; 3 Department of Family Medicine, Seoul National University Hospital, Seoul National University College of Medicine, 101 Daehak-Ro, Jongno-Gu, Seoul, South Korea; 4 Department of Family Medicine, Chung-Ang University Gwangmyeong Hospital, Chung-Ang University College of Medicine, Gwangmyeong-si, South Korea; 5 Home-Based Medical Care Team, Public Healthcare Center, Seoul National University Hospital, 101 Daehak-Ro, Jongno-Gu, Seoul, South Korea; 6 Department of Family Medicine, Gachon University Gil Medical Center, 21, Namdong-daero 774 beon-gil, Namdong-gu, Incheon, South Korea

**Keywords:** Cardiology, Risk factors

## Abstract

Hemoglobin variability is known to increase cardiovascular mortality in chronic kidney disease, but the association of hemoglobin variability with the risk of cardiovascular disease (CVD) in the general population is yet unclear. This retrospective cohort study based on ‘the South Korean National Health Insurance Service database’ consisted of 198,347 adults who went through all three health examinations. Hemoglobin variability is defined as the average successive variability of three separate hemoglobin values from each health screening period. Participants were followed up for 6 years to determine the risk of coronary heart disease and stroke. We used multivariate Cox proportional hazards regression to examine the adjusted hazard ratios for CVD according to hemoglobin variability. Per 1 unit increase of hemoglobin variability, the risk for CVD (aHR 1.06, 95% CI 1.02–1.09) and stroke (aHR 1.08, 95% CI 1.03–1.13) increased significantly. The risk-increasing trend was preserved in the low-to-moderate risk group of CVDs (aHR 1.07, 95% CI 1.02–1.11). This result suggests that subjects with high hemoglobin variability who would otherwise be categorized as having low-to-moderate CVD risk may have higher risk of CVD than those with low hemoglobin variability.

## Introduction

The Global Burden of Disease study reported that cardiovascular diseases (CVDs) are one of the primary causes of death worldwide^1^. Although the CVD mortality in high-income countries has been decreased for the past half-century, the decline rate has slowed down^2^. In addition, those who survived from cardiovascular event will still experience serious sequela such as arrhythmias or physical disability. Thus, it is important to prevent CVD by identifying and managing CVD risk factors prior to cardiovascular event development. Metabolic parameters such as cholesterol level^3^, glucose level^4^, blood pressure^5^, and body weight^6^ are well-known risk factors of CVD. Moreover, the variability of such parameters over time, which reflects the dysregulation of homeostasis, has also previously been shown to be associated with the increased risk of CVD^7–11^. The variability of hemoglobin levels has been demonstrated to be associated with the increased cardiovascular mortality, only in patients with chronic kidney disease (CKD)^12^. However, hemoglobin variability in the general population is not fully studied as a potential risk factor of CVD, while low hemoglobin concentration and change in hemoglobin levels were shown to be risk factors for CVD^13,14^.

In the general population, high hemoglobin variability is related to the increased all-cause mortality^15^. Another study examined that high hemoglobin variability is associated with higher risk of hypertension in the general population^16^. Since the leading cause of mortality is CVD globally^1^, and that hypertension is one of the main risk factors of CVD^5^, it is reasonable to assume that the risk of CVD also may be elevated with high hemoglobin variability. In fact, hemoglobin variability reflects the inflammatory state and repeated anemic stimuli which are considered to increase the risk of CVDs by triggering atherosclerosis and ventricular hypertrophy, respectively^17,18^. Thus, there is a need for more studies about the impact of hemoglobin variability on subsequent CVD outcomes.

We, therefore, aimed to determine the association between hemoglobin variability and the risk of CVD using the National Health Insurance Service (NHIS) database from South Korea.

## Methods

### Study design

This retrospective cohort study is based on the National Health Insurance Service—National Health Screening Cohort (NHIS-HEALS) database during 2002–2013. The national health screening for South Korean aged 40 years or more is performed biannually by the National Health Insurance which covers 97% of all citizens of South Korea^19^. The health examination includes basic physical examinations assessing the body mass index (BMI) and blood pressure, blood test detecting biochemical indicators such as total cholesterol, fasting serum glucose (FSG), and hemoglobin concentration, and self-reported questionnaires consisted of multiple-choice questions regarding health behavior and medical history. To construct the NHIS-HEALS database, the NHIS used the simple random sampling method and combined health examination information with clinical information such as hospital use, diagnoses, and death. Numerous epidemiological studies have used this database previously, and its applicability has been demonstrated elsewhere^20^.

### Study population

**Figure 1:**
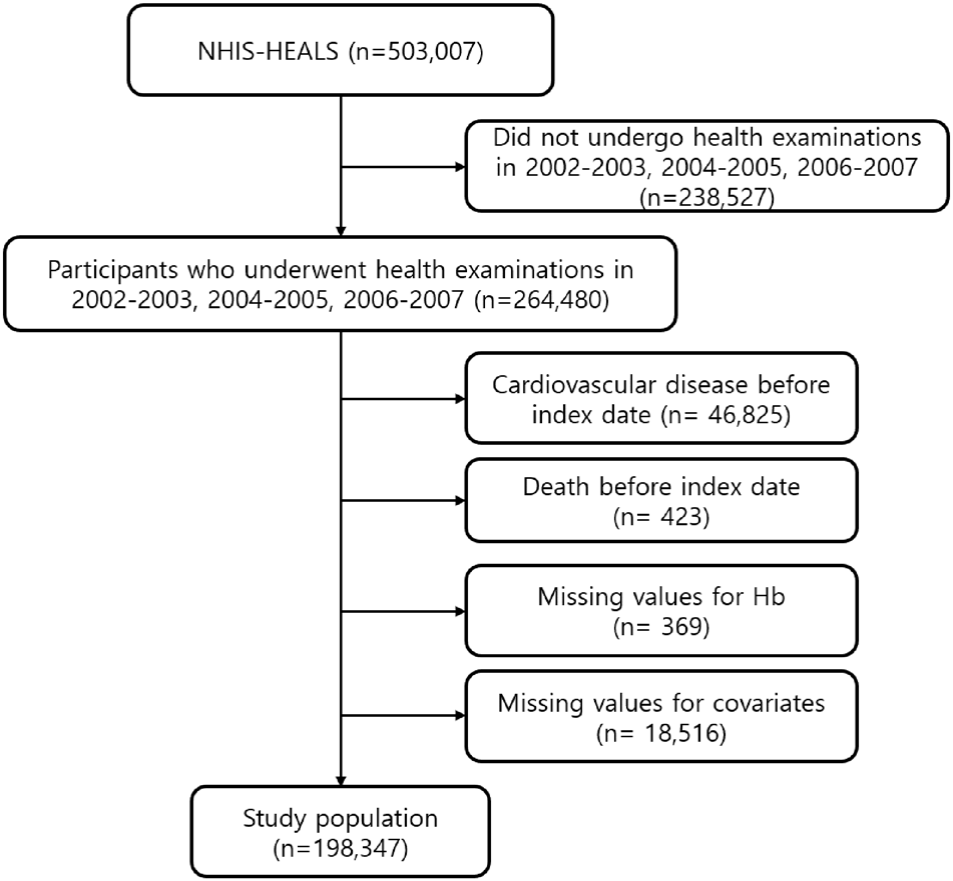
Flowchart for the inclusion of study population.

Among 264,480 subjects over 40 years old who went through all three health examinations in the first (2002–2003), second (2004–2005), and third (2006–2007) periods, 47,248 individuals who were already diagnosed with CVD or died before the index date of 1 January 2008 were excluded (Fig. 1). In addition, we excluded 369 participants who did not have values for hemoglobin levels, and 18,516 participants who lacked information on other covariates. A total of 198,347 participants were included in this study.

### Ethical approval

The study protocol conformed to the ethical guidelines of the 1975 Declaration of Helsinki. This study was approved by the Seoul National University Hospital Institutional Review Board (IRB number: 1703-039-836). The requirement for informed consent was waived as the NHIS database was anonymized according to strict confidentiality guidelines prior to distribution.

### Data use

The data from this study is directly available via the NHIS database registration system and thus cannot be allowed for other researchers to access when they are intended to replicate the procedures.

### Exposure: hemoglobin variability

We calculated hemoglobin variability using the average successive variability (ASV) method^21,22^. Firstly, we took hemoglobin values from three health examinations. Then we subtracted the values from second to first examination, and third to second examination to get the differences. We calculated the average of those two differences of hemoglobin values.

### Outcome: cardiovascular disease

The primary outcome of this study is CVDs which is divided into coronary heart diseases (CHDs) and strokes according to the American Heart Association (AHA) guidelines^23^. We defined CVD events as hospitalization for 2 days or more with CHDs or strokes from January 1, 2008 to December 31, 2013 to minimize the possibility of misdiagnoses. We used the International Classification of Diseases, Tenth Revision (ICD-10) codes to identify CHDs (I20–I25) and strokes (I60–I69). Previous studies suggest that it is accurate to define CVD based on the ICD-10 code^24^.

### Covariates

We included sex, age, household income (first, second, third, or fourth quartiles), initial hemoglobin concentration, change in hemoglobin levels, smoking state (never, pass, or current smoker), alcohol consumption (0, 1–2, 3–4, 5–6, or 7 times per week), Charlson comorbidity index (CCI), systolic blood pressure, total cholesterol, and BMI as possible confounding covariates. The initial hemoglobin concentration is the hemoglobin value from the first examination, and the change in hemoglobin levels was calculated by subtracting hemoglobin levels of the first examination from those of the third examination. We divided body weight by height squared to calculate BMI, and household income was categorized by insurance premium. CCI is the most extensively used comorbidity index^25^, which includes the risk of various comorbidities such as kidney disease, chronic lung disease, liver disease, dementia, and malignant tumors^26^. We also used the European Society of Cardiology (ESC) Systemic Coronary Risk Estimation (SCORE) for a subgroup analysis. It is a reasonable tool recommended by the ESC to estimate an individual’s 10-year risk of future fatal CVD using age, sex, systolic blood pressure, smoking state, and total cholesterol level^29^. We used the low-risk SCORE chart which is established for the European countries of which 2016 CVD mortality rate was under 150 per 100,000, based on the 2016 age-adjusted CVD mortality in WHO data from the Global Burden of Disease Study^29,30^.

### Statistical analysis

We used Multivariate cox proportional hazards regression to examine the adjusted hazard ratios (aHRs) and confidence intervals (CIs) of 95% for CVDs, CHDs and strokes. We considered the p value under 0.05 as statistically significant. We also used restricted cubic spline regression model to present the continuous relationship between hemoglobin variability and the aHRs of CVD risk^27^. Referring to previous studies, we placed 4 knots at the 5th, 35th, 65th, 95th percentiles of hemoglobin variability^15,28^. Considering potential bias due to other causes before the follow-up investigation, 1- and 2-year of latent periods were washed out for sensitivity analyses. In addition, we performed stratified analyses according to several confounding factors: age, physical activity, smoking state, alcohol consumption, CCI, hemoglobin concentration, and change in hemoglobin levels. We also performed subgroup analyses according to the ESC SCORE. Individuals who got ESC SCORE lower than 5% are considered as low-to-moderate risk groups for future fatal CVD, according to the European Atherosclerosis Society (EAS) and ESC guidelines^29^. Data collection was performed by SAS Enterprise Guide 7.1 (SAS Institute, Cary, NC, USA), and conducted all statistical analyses by STATA version 13 (StataCorp, College Station, Texas, USA).

## Results

Among the total cohort of 198,347, the number (%) of men and women were 116,016 (58.5) and 82,331 (41.5). The average follow-up duration of total cohort is 5.8 years. Baseline characteristics of total cohort, men and women are demonstrated in Table 1. The mean age [standard deviation (SD)] of total cohort is 55.3 (8.5) years. The mean value (SD) of hemoglobin variability (ASV), initial hemoglobin concentration, and change of hemoglobin levels of total cohort were 0.9 (0.6) g/dL, 14.0 (1.5) g/dL, and 0.2 (1.2) g/dL, respectively. The mean (SD) BMI was 23.9 (2.8) kg/m^2^; systolic blood pressure, 125.1 (15.9) mmHg; FSG, 97.6 (24.8) mg/dL; and total cholesterol, 198.9 (36.2) mg/dL. Among total participants, 39% were categorized as the highest quartile of household income, 47% reported that they do not exercised, and 68.7% had 1 or lower CCI scores. More than half of the participants were reported as non-alcohol consumers (56.9%) and never smokers (70.6%).

**Table 1:** Descriptive characteristics of the study population.

	Total	Men	Women	*p*-value^a^
Number of people (%)	198,347	116,016 (58.5)	82,331 (41.5)	
Age, years, mean (SD)	55.3 (8.5)	54.7 (8.3)	56.2 (8.6)	< 0.001
Hemoglobin variability (ASV), g/dL, mean (SD)	0.9 (0.6)	0.8 (0.6)	0.9 (0.6)	< 0.001
Initial Hemoglobin concentration, g/dL, mean (SD)	14.0 (1.5)	14.9 (1.1)	12.8 (1.2)	< 0.001
Change in Hemoglobin concentration, g/dL, mean (SD)	0.2 (1.2)	0.1 (1.2)	0.0 (1.3)	< 0.001
Household income, N (%)				< 0.001
1st (highest)	77,358 (39.0)	52,118 (44.9)	25,240 (30.7)	
2nd	56,695 (28.6)	34,205 (29.5)	22,490 (27.3)	
3rd	39,334 (19.8)	18,574 (16.0)	20,760 (25.2)	
4th (lowest)	24,960 (12.6)	11,119 (9.6)	13,841 (16.8)	
Smoking, N (%)				< 0.001
Never smoker	139,885 (70.6)	59,028 (50.9)	80,857 (98.2)	
Past smoker	18,677 (9.4)	18,227 (15.7)	450 (0.6)	
Current smoker	39,785 (20.1)	38,761 (33.4)	1024 (1.2)	
Alcohol consumption, times per week, N (%)				< 0.001
0	112,864 (56.9)	43,846 (37.8)	69,018 (83.8)	
< 1	30,908 (15.6)	22,617 (19.5)	8291 (10.1)	
1–2	35,722 (18.0)	31,750 (27.4)	3972 (4.8)	
3–4	12,522 (6.3)	11,896 (10.3)	626 (0.8)	
≥ 5	6331 (3.2)	5907 (5.1)	424 (0.5)	
Physical activity, times per week, N (%)				< 0.001
0	93,245 (47.0)	47,077 (40.6)	46,168 (56.1)	
1–2	58,676 (29.6)	40,600 (35.0)	18,076 (22.0)	
3–4	26,882 (13.6)	17,106 (14.7)	9776 (11.9)	
5–6	6727 (3.4)	3963 (3.4)	2764 (3.4)	
7	12,817 (6.5)	7270 (6.3)	5547 (6.7)	
Body mass index, kg/m^2^, mean (SD)	23.9 (2.8)	24.0 (2.7)	23.7 (2.9)	< 0.001
Systolic blood pressure, mmHg, mean (SD)	125.1 (15.9)	126.6 (15.3)	123.1 (16.5)	< 0.001
Fasting serum glucose, mg/dL, mean (SD)	97.6 (24.8)	99.7 (26.7)	94.6 (21.5)	< 0.001
Total cholesterol, mg/dL, mean (SD)	198.9 (36.2)	196.5 (35.2)	202.4 (37.2)	< 0.001
Charlson comorbidity index, N (%)				< 0.001
0–1	136,343 (68.7)	84,513 (72.9)	51,830 (63.0)	
2–3	50,997 (25.7)	25,870 (22.3)	25,127 (30.5)	
≥ 4	11,007 (5.6)	5633 (4.9)	5374 (6.5)	
ESC score				< 0.001
Low-to-moderate risk group	180,695 (91.0)	99,064 (85.39)	81,631 (99.15)	
High risk group	17,652 (8.9)	16,951 (14.61)	700 (0.85)	

*ASV* average successive variability, *SD* standard deviation, *N* number of people.

^a^*p*-value calculated by chi-square test or t-test according to variables.

During follow-up duration, CVD, CHD, stroke events each occurred 8687 (4.4%), 4001 (2.0%), and 4744 (2.4%) in the total cohort. The numbers of CVD, CHD, stroke events per each year in the follow-up duration are shown in the Supplementary Table S1. The association between hemoglobin variability and the risk of CVD, CHD and stroke is shown in Table 2. The risk of CVD was elevated per 1 unit increase of hemoglobin variability for the total cohort (aHR 1.06, 95% CI 1.02–1.09) as well as for men (aHR 1.06, 95% CI 1.02–1.11) and women (aHR 1.06, 95% CI 1.00–1.12). The risk of stroke also increased in total cohort (aHR 1.08, 95% CI 1.03–1.13), men (aHR 1.07, 95% CI 1.01–1.14) and women (aHR 1.08, 95% CI 1.01–1.16) per 1 unit increase of hemoglobin variability. The risk-increasing trends of CVD and stroke were preserved in sensitivity analyses after washing out 1-year latent period (see Supplementary Table S2). However, the risk-increasing trend of CHD was not statistically significant.

**Table 2:** Association of hemoglobin variability on cardiovascular disease (coronary heart disease and stroke).

	Total	Men	Women	*p* for interaction*
Cardiovascular disease				
Events (%)	8687 (4.4)	5568 (4.8)	3119 (3.8)	
aHR (95% CI)	1.06 (1.02–1.09)	1.06 (1.02–1.11)	1.06 (1.00–1.12)	0.956
Coronary heart disease				
Events (%)	4001 (2.0)	2770 (2.4)	1231 (1.5)	
aHR (95% CI)	1.04 (0.99–1.09)	1.04 (0.98–1.11)	1.03 (0.95–1.13)	0.994
Stroke				
Events (%)	4744 (2.4)	2839 (2.5)	1905 (2.3)	
aHR (95% CI)	1.08 (1.03–1.13)	1.07 (1.01–1.14)	1.08 (1.01–1.16)	0.935

Hazard ratio calculated by Cox proportional hazards regression analysis after adjustments for age, sex, initial hemoglobin level, change in hemoglobin level, household income, smoking, alcohol consumption, physical activity, systolic blood pressure, fasting serum glucose, total cholesterol, and Charlson comorbidity index.

*aHR* adjusted hazard ratio, *CI* confidence interval.

*p value for interaction between men and women.

The aHRs of the covariates for CVD are provided in Supplementary Table S3. Among various confounders, age, initial hemoglobin level, income, smoking state, BMI and CCI showed relatively strong contribution to CVD risk.

**Figure 2:**
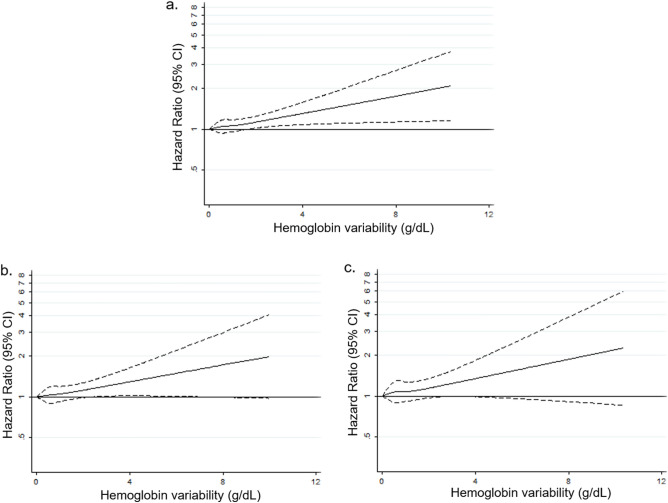
Restricted cubic spline graph of hazard ratio for cardiovascular disease in total cohort (**a**), men (**b**) and women (**c**). Hazard ratio is calculated by Cox proportional hazards regression analysis after adjustments for age, sex, initial hemoglobin level, change in hemoglobin level, household income, smoking, alcohol consumption, physical activity, systolic blood pressure, fasting serum glucose, total cholesterol, and Charlson comorbidity index. *CI* confidence interval.

**Figure 3:**
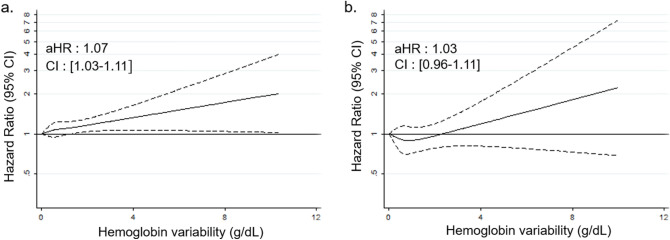
Restricted cubic spline graph of hazard ratio for cardiovascular disease in the subgroups of ESC SCORE : Low-to-moderate risk group (**a**) and High risk group (**b**). Hazard ratio is calculated by Cox proportional hazards regression analysis after adjustments for age, sex, initial hemoglobin level, change in hemoglobin level, household income, smoking, alcohol consumption, physical activity, systolic blood pressure, fasting serum glucose, total cholesterol, and Charlson comorbidity index. P value for interaction between the subgroups is 0. 434. *ESC SCORE* European Society of Cardiology Systemic Coronary Risk Estimation, *aHR* adjusted hazard ratio, *CI* confidence interval.

The association of hemoglobin variability with the risk of CVD is plotted by restricted cubic spline regression in Fig. 2. The higher hemoglobin variability was related to the increased risk of CVD in a dose-responsive manner for total cohort as well as in both men and women. We also plotted Restricted cubic spline graph to show the result of subgroup analyses by ESC SCORE in Fig. 3. Higher hemoglobin variability was significantly associated with the elevated risk of CVD for the low-to-moderate risk group (aHR 1.07, 95% CI 1.02–1.11).

Results from stratified analyses according to the age, physical activity, smoking state, alcohol consumption, CCI, hemoglobin concentration, and change direction in hemoglobin levels are demonstrated in Table 3. As the hemoglobin variability increases, the risk of CVD elevated in both 60 years or older (aHR 1.07, 95% CI 1.02–1.12) and younger than 60 years old (aHR 1.05, 95% CI 1.00–1.10). When stratified by physical activity, smoking state, and alcohol consumption, one unit increase of hemoglobin variability was associated with the elevated risk of CVD in all subgroups: never smokers (aHR 1.06, 95% CI 1.02–1.11), ever smokers (aHR 1.06, 95% CI 1.00–1.12), non-alcohol consumers (aHR 1.06, 95% CI 1.02–1.11), and alcohol consumers (aHR 1.05, 95% CI 1.00–1.11). Both participants who got CCI scores of 0–1 (aHR 1.07, 95% CI 1.03–1.12) and those with CCI scores ≥ 2 (aHR 1.05, 95% CI 1.00–1.11) showed increased risk of CVD in the total cohort. When divided by whether participants had the hemoglobin concentration of anemia at least once among three health examinations or not, only those without anemia at all showed significantly higher risk of CVD (aHR 1.07, 95% CI 1.02–1.11). When stratified by the direction of change between hemoglobin levels, only those who had increased hemoglobin concentration in the third examination compared to the first examination showed significantly elevated risk of CVD (aHR 1.07, 95% CI 1.02–1.12). However, all of the p-values for interaction between subgroups of each confounder were not statistically significant.

**Table 3:** Stratified analyses on the association of hemoglobin variability with cardiovascular disease risk according to subgroups of age, initial hemoglobin level, change in hemoglobin levels, smoking state, physical activity, alcohol consumption, Charlson comorbidity index and ESC SCORE.

	Adjusted hazard ratio (95% confidence interval)			
	Total	Men	Women	*p* for interaction*
Age				
< 60	1.07 (1.02–1.12)	1.08 (1.02–1.15)	1.05 (0.97–1.14)	0.538
≥ 60	1.05 (1.00–1.10)	1.04 (0.98–1.10)	1.07 (0.99–1.16)	
Physical activity				
Inactive	1.06 (1.01–1.11)	1.06 (1.00–1.13)	1.05 (0.98–1.13)	0.973
Active	1.06 (1.01–1.11)	1.06 (1.00–1.12)	1.06 (0.96–1.16)	
Smoking state				
Never smoker	1.06 (1.02–1.11)	1.07 (1.01–1.13)	1.06 (1.00–1.12)	0.655
Ever smoker	1.06 (1.00–1.12)	1.05 (0.99–1.12)	1.13 (0.81–1.58)	
Alcohol consumption				
Non-consumer	1.06 (1.02–1.11)	1.06 (0.99–1.13)	1.07 (1.00–1.13)	0.852
Consumer	1.05 (1.00–1.11)	1.06 (1.00–1.12)	1.01 (0.86–1.19)	
Charlson comorbidity index				
0–1	1.07 (1.03–1.12)	1.07 (1.01–1.13)	1.09 (1.00–1.18)	0.344
≥ 2	1.05 (1.00–1.11)	1.05 (0.99–1.13)	1.05 (0.97–1.13)	
Hemoglobin concentration				
No Anemia^a^	1.07 (1.02–1.11)	1.05 (0.99–1.10)	1.12 (1.03–1.22)	0.581
Anemia^b^	1.05 (0.99–1.11)	1.05 (0.97–1.14)	1.04 (0.96–1.13)	
Change in hemoglobin levels^c^				
Decrease	1.04 (0.99–1.10)	1.05 (0.98–1.12)	1.04 (0.96–1.13)	0.875
Increase	1.07 (1.02–1.12)	1.06 (1.00–1.12)	1.08 (1.00–1.16)	
ESC score				
Low-to-moderate risk group	1.07 (1.03–1.11)	1.07 (1.02–1.13)	1.06 (1.00–1.12)	0.434
High risk group	1.03 (0.96–1.11)	1.03 (0.96–1.11)	1.01 (0.70–1.45)	

Hazard ratio calculated by Cox proportional hazards regression analysis after adjustments for age, sex, initial hemoglobin level, change in hemoglobin level, household income, smoking, alcohol consumption, physical activity, systolic blood pressure, fasting serum glucose, total cholesterol, and Charlson comorbidity index.

*p value for interaction between subgroups in total cohort.

^a^No anemia in all three health examinations.

^b^Anemia at least once among three health examinations.

^c^The direction of change between the hemoglobin concentration in first health examination with that in third health examination of each individuals.

## Discussion

In this retrospective cohort study, we observed that high hemoglobin variability was significantly associated with increased risk of CVD. To our knowledge, this was the first large population-based study on the association of hemoglobin variability with the risk of CVD in the general population.

Most previous studies regarding hemoglobin variability focused on patients with CKD^12,31^. One study examined that 1 SD increase of hemoglobin variability in hemodialysis patients was associated with a 10% increase in cardiovascular death rate^12^. With regard to the general population, there is a study which determined that 5% SD elevation of hemoglobin variability was related to 8% increase in all-cause mortality^15^. In another study using the general population, the highest hemoglobin variability group had 9% higher hazard ratio of hypertension than the lowest group^16^. In this study, we observed that 1 unit elevation of hemoglobin variability was related to the higher risk of CVDs in the general population.

Hemoglobin concentration or its variability may be affected by various factors including demographic characteristics, such as gender, age^32^, behavioral factors, such as exercise^33^, and, smoking^34^, biochemical indicators, such as plasma volume^35^, medications, such as iron preparations^36^, and erythropoiesis-stimulating agents^37,38^, and clinical state, such as inflammation, infection^39^, and malignancies^40^. Among those factors, there are several possible mechanisms to explain how high hemoglobin variability could increase the risk of CVD. First, high hemoglobin variability can cause left ventricular hypertrophy (LVH)^15,41^. In chronic anemia, the growth of myocardial cell is stimulated and cardiac output increases as compensation for the decreased number of red blood cells^41^. When there is high variability in hemoglobin levels, anemic stimuli would be repeated, which can eventually cause enlargement of ventricles^15^. LVH is proved to be an accurate marker of CVD^42^, and also has been examined to be a prognostic factor independent of hypertension^43^. Second, hemoglobin variability may be a marker of underlying active inflammatory response, which is related to an elevated risk of CVD. Inflammatory cytokines like Tumor Necrosis Factor-alpha (TNF-α) and Interferon-γ (IFN-γ) can cause high hemoglobin variability by interfering the differentiation of erythroid progenitor cells and the production of erythropoietin in the kidneys, and limiting iron availability for erythrocytes^18^. Inflammation plays an important role throughout all stages of atherosclerosis which is in turn closely related with cardiovascular risks^17^.

To assess the possible confounding effect of behavioral factors, including alcohol consumption, physical activity, and smoking state, age, and comorbidities, we performed stratified analyses according to the subgroups of those factors. Previous studies found that CKD patients with higher hemoglobin concentration were more likely to be physically active than those with lower concentration^44^. Physical activity is associated with the decreased risk of CHD^45^ and stroke^46^, which decreases the risk of CVD by lowering other risk factors like inflammation and blood pressure^47^. Additionally, smoking state can also alter the hemoglobin concentration^34^. In all subgroups of the total cohort, higher hemoglobin variability was related to the increased risk of CVD regardless of such confounding factors. Age is also considered to be a major risk factor of CVD^48^, and hemoglobin level tends to decrease as age increases, especially in men^49^. When stratified by age, the risk-increasing trend was also preserved in all subgroups of total cohort. Comorbidities such as chronic renal disease is also a well-known risk factor of CVD events^50^. To take this into account, we used Charlson comorbidity index (CCI) which is a measure of overall comorbidity status^25^. We also found that the risk-increasing association was preserved despite the subgroups of CCI in total cohort.

In previous studies, the risk of CVD can be elevated by the underlying anemia and the change of hemoglobin levels^13,14^. Thus, we conducted stratified analysis by whether participants had hemoglobin concentration of anemia at least once among three health examinations or not, and by the change direction in hemoglobin levels. Although only subgroups of anemia and increasing hemoglobin group showed significantly increasing hazard ratios, we can expect that hemoglobin variability could have an independent effect on CVD excluding the effect of anemia and hemoglobin change since p-values for interaction between subgroups were not statistically significant. These results of subgroup analyses suggest that hemoglobin variability could be a meaningful risk factor of CVD regardless of other confounders.

To further investigate the clinical implications of hemoglobin variability among generally healthy populations, we conducted a subgroup analysis of those who were low-to-moderate risk according to the ESC SCORE which predicts 10-year risk of future fatal CVD^29^. High hemoglobin variability was significantly associated with increased risk of CVD even in the low-to-moderate risk group of ESC SCORE. Compared to the high risk population who have many other screening clinical indicators like high blood pressure or serum lipid level, there is a relative lack of effective predictors of the CVD risk for low-to-moderate risk population. On the basis of this subgroup analysis, we can consider hemoglobin variability as a useful biomarker for stratifying the risk of CVD among the low-to-moderate risk group classified by a well-established risk score.

There are several limitations in this study. First, the risk of CVD could be underestimated due to the operational definition of CVD events. We defined the incidence of CVD as hospitalization for 2 days or more with coronary heart diseases or strokes to minimize the possibility of misdiagnosis with reference to previous studies regarding CVD events^51–53^. Although this definition is still missing the sudden death from cardiovascular diseases which imply fatal CVD cases, the risk-increasing trend was significant despite the possibility of underestimation. Second, this study has the probability of selection bias by limiting the study population to those who took all three health examinations in the given period. To quantify the effect of selection bias, we compared descriptive characteristics such as age, sex, income and CCI between the study population and the others who did not take all three health examinations (see Supplementary Table S4). P-values show that those descriptive characteristics are significantly different between those two groups, and this result implies the possibility of selection bias. Therefore, we need further studies to find out the association of hemoglobin variability and CVD in the excluded group. Third, the database we used was missing several confounders of which the association with hemoglobin concentration or the risk of CVD is proved by previous studies. It is well known that inflammation can alter hemoglobin levels, but we could not detect inflammatory markers such as C-reactive protein (CRP) or erythrocyte sedimentation rate (ESR)^18^. Hemoglobin concentration can be also affected by various coexisting diseases, including CKD^54^ and chronic obstructive pulmonary disease^55^. Although we could not adjust all of the comorbidities separately, we used CCI in which the risk of chronic pulmonary disease, renal disease, and other various comorbidities are considered^26^. In fact, we found that high hemoglobin variability is associated with increased risk of CVD even in the subgroup of CCI values of 2 or less, in which most chronic diseases were excluded. Further studies adjusting these missing covariates are necessary to prove the independent effect of hemoglobin variability on the risk of CVD. Fourth, since the study design is retrospective cohort, additional studies are needed to prove the exact causal relationship between hemoglobin variability and CVD. Finally, since the study population is 40 years or older South Korean adults, this study may not be enough for generalization to other countries.

In conclusion, individuals with high hemoglobin variability are more likely to suffer from CVD in the future, compared to those with low variability. Hemoglobin variability may be a meaningful risk factor of CVD in the general population. Specifically, when individuals who were categorized as the low-to-moderate CVD risk group by well-established CVD risk assessment scores show high hemoglobin variability, it may be beneficial to closely follow-up these subjects for early signs and symptoms of CVD.

## Supplementary Information

Supplementary Tables.

## Data Availability

The datasets generated and/or analyzed during the current study are available from the corresponding author on reasonable request.
