# Supplementary material for: Association of hemoglobin variability with the risk of cardiovascular disease: a nationally representative retrospective cohort study from South Korea
Source: Sci Rep. 2023 Feb 7;13:2148. doi: 10.1038/s41598-023-28029-w (PMC9905090; doi:10.1038/s41598-023-28029-w)
Supplement: Supplementary file 1 — Supplementary Tables. [file 41598_2023_28029_MOESM1_ESM.docx]

**Supplementary Information**

**Supplementary Table S1. The number of CVD outcomes per year in the follow-up duration.**

| **Number of people (%)** | **CVD** | **CHD** | **Stroke** |
| --- | --- | --- | --- |
| **2008** | 1,230 (14.2) | 573 (14.3) | 665 (14.0) |
| **2009** | 1,385 (15.9) | 646 (16.1) | 756 (15.9) |
| **2010** | 1,426 (16.4) | 692 (17.3) | 738 (15.6) |
| **2011** | 1,513 (17.4) | 686 (17.1) | 841 (17.7) |
| **2012** | 1,550 (17.8) | 712 (17.8) | 844 (17.8) |
| **2013** | 1,583 (18.2) | 692 (17.3) | 900 (19.0) |
| **Total** | 8,687 | 4,001 | 4,744 |

**Supplementary Table S2. Sensitivity Analyses on Association of Hemoglobin and CVD after washing out latent periods**

|  | **CVD** | **CHD** | **Stroke** |
| --- | --- | --- | --- |
| **1 Year washed out** | | | |
| **Events (%)** | 7,457 (3.8) | 3,428 (1.7) | 4,079 (2.1) |
| **aHR (95% CI)^a^** | 1.06 (1.02-1.09) | 1.03(0.97-1.09) | 1.08 (1.03-1.13) |
| **2 Year washed out** | | | |
| **Events (%)** | 6,072 (3.1) | 2,782 (1.4) | 3,323 (1.7) |
| **aHR (95% CI)^a^** | 1.03 (0.99-1.07) | 1.01 (0.95-1.07) | 1.04 (0.99-1.10) |

^a^Hazard ratio calculated by Cox proportional hazards regression analysis after adjustments for age, sex, initial hemoglobin level, change in hemoglobin level, household income, smoking, alcohol consumption, physical activity, systolic blood pressure, fasting serum glucose, total cholesterol, and Charlson comorbidity index.

Acronyms: HR, hazard ratio; CI, confidence interval.

**Supplementary Table S3. Hazard Ratios of Covariates for Coronary Heart Disease.**

|  | aHR (95% CI)^a^ | | |
| --- | --- | --- | --- |
| **Covariate** | Total | Men | Women |
| Age | 1.06 (1.06-1.07) | 1.06 (1.06-1.07) | 1.07 (1.06-1.07) |
| Initial Hemoglobin concentration | 1.02 (1.00-1.04) | 1.01 (0.99-1.04) | 1.03 (0.99-1.07) |
| Change in Hemoglobin concentration | 1.00 (0.98-1.02) | 1.00 (0.98-1.03) | 0.99 (0.95-1.02) |
| Household income | | | |
| 1^st^ (highest) | 1.00 (reference) | 1.00 (reference) | 1.00 (reference) |
| 2^nd^ | 1.03 (0.98-1.09) | 1.00 (0.94-1.07) | 1.09 (0.99-1.19) |
| 3^rd^ | 1.03 (0.97-1.09) | 1.02 (0.95-1.10) | 1.05 (0.95-1.15) |
| 4^th^ (lowest) | 1.07 (1.00-1.14) | 1.06 (0.97-1.16) | 1.08 (0.97-1.20) |
| Smoking | | | |
| Never smoker | 1.00 (reference) | 1.00 (reference) | 1.00 (reference) |
| Past smoker | 1.09 (1.01-1.18) | 1.06 (0.98-1.16) | 2.16 (1.51-3.09) |
| Current smoker | 1.53 (1.44-1.62) | 1.53 (1.44-1.62) | 1.36 (1.06-1.76) |
| Alcohol consumption, times per week | | | |
| 0 | 1.00 (reference) | 1.00 (reference) | 1.00 (reference) |
| <1 | 0.98 (0.91-1.05) | 0.97 (0.90-1.05) | 0.94 (0.81-1.08) |
| 1-2 | 0.86 (0.80-0.92) | 0.84 (0.78-0.90) | 0.97 (0.80-1.17) |
| 3-4 | 0.87 (0.79-0.95) | 0.87 (0.79-0.96) | 0.60 (0.35-1.04) |
| >=5 | 0.91 (0.82-1.01) | 0.88 (0.79-0.99) | 1.42 (0.96-2.09) |
| Physical activity, times per week | | | |
| 0 | 1.00 (reference) | 1.00 (reference) | 1.00 (reference) |
| 1-2 | 0.92 (0.87-0.97) | 0.95 (0.89-1.01) | 0.85 (0.77-0.94) |
| 3-4 | 0.95 (0.89-1.02) | 1.00 (0.92-1.09) | 0.86 (0.75-0.97) |
| 5-6 | 0.86 (0.76-0.98) | 0.82 (0.70-0.96) | 0.97 (0.79-1.19) |
| 7 | 0.97 (0.89-1.05) | 0.98 (0.89-1.09) | 0.95 (0.83-1.08) |
| Body mass index | 1.03 (1.02-1.03) | 1.02 (1.01-1.03) | 1.03 (1.02-1.04) |
| Systolic blood pressure | 1.01 (1.01-1.01) | 1.01 (1.01-1.01) | 1.01 (1.01-1.01) |
| Fasting serum glucose | 1.00 (1.00-1.00) | 1.00 (1.00-1.00) | 1.00 (1.00-1.00) |
| Total cholesterol | 1.00 (1.00-1.00) | 1.00 (1.00-1.00) | 1.00 (1.00-1.00) |
| Charlson comorbidity index | 1.12 (0.10-1.13) | 1.11 (1.09-1.13) | 1.13 (1.10-1.15) |

^a^Hazard ratio calculated by Cox proportional hazards regression analysis after adjustments for age, sex, initial hemoglobin level, change in hemoglobin level, household income, smoking, alcohol consumption, physical activity, systolic blood pressure, fasting serum glucose, total cholesterol, and Charlson comorbidity index.

Acronyms: HR, hazard ratio; CI, confidence interval.

**Supplementary Table S4. Comparison of descriptive characteristics between the study population and exclusion group.**

|  | Study population^a^ | Excluded group^b^ | *p-value^c^* |
| --- | --- | --- | --- |
| Number of people (%) | 264,480 (52.6) | 238,527 (47.4) |  |
| Age, years, mean (SD) | 56.4 (8.9) | 58.6 (10.0) | <0.001 |
| Sex, Number of people (%) | | | <0.001 |
| Men | 155,684 (58.9) | 115,309 (48.3) |  |
| Women | 108,796 (41.1) | 123,218 (51.7) |  |
| Household income, N (%) | | | <0.001 |
| 1^st^ (highest) | 102,975 (38.9) | 69,882 (29.3) |  |
| 2^nd^ | 75,482 (28.5) | 71,605 (30.0) |  |
| 3^rd^ | 52,319 (19.8) | 54,338 (22.8) |  |
| 4^th^ (lowest) | 33,704 (12.7) | 42,702 (17.9)) |  |
| Charlson comorbidity index, N (%) | | | <0.001 |
| 0-1 | 165,172 (62.5) | 137,620 (57.7) |  |
| 2-3 | 76,047 (28.8) | 72,233 (30.3) |  |
| ≥4 | 23,261 (8.8) | 28,674 (12.0) |  |

^a^Study population is a group who took all three health examinations in the give period.

^b^Excluded group is a group who did not take all three health examinations.

^c^p-value is calculated by chi-square test or t- test according to variables.

Acronyms: SD, standard deviation; N, number of people.
